# A Genome-Wide CRISPR/Cas9 Screen Reveals the Requirement of Host Sphingomyelin Synthase 1 for Infection with Pseudorabies Virus Mutant gD^–^Pass

**DOI:** 10.3390/v13081574

**Published:** 2021-08-09

**Authors:** Julia E. Hölper, Finn Grey, John Kenneth Baillie, Tim Regan, Nicholas J. Parkinson, Dirk Höper, Thiprampai Thamamongood, Martin Schwemmle, Katrin Pannhorst, Lisa Wendt, Thomas C. Mettenleiter, Barbara G. Klupp

**Affiliations:** 1 Institute of Molecular Virology and Cell Biology, Friedrich-Loeffler-Institut, 17493 Greifswald, Insel Riems, Germany; julia.hoelper@fli.de (J.E.H.); katrin.pannhorst@fli.de (K.P.); lisa.wendt@fli.de (L.W.); thomasc.mettenleiter@fli.de (T.C.M.); 2 The Roslin Institute, University of Edinburgh, Easter Bush, Midlothian EH25 9RG, UK; fgrey@exseed.ed.ac.uk (F.G.); j.k.baillie@ed.ac.uk (J.K.B.); Tim.Regan@roslin.ed.ac.uk (T.R.); nick.parkinson@ed.ac.uk (N.J.P.); 3 Intensive Care Unit, Royal Infirmary of Edinburgh, Edinburgh EH25 9RG, UK; 4 Institute of Diagnostic Virology, Friedrich-Loeffler-Institut, 17493 Greifswald, Insel Riems, Germany; dirk.hoeper@fli.de; 5 Institute of Virology, Medical Center-University of Freiburg, 79110 Freiburg, Germany; thiprampai@gmail.com (T.T.); martin.schwemmle@uniklinik-freiburg.de (M.S.); 6 Spemann Graduate School of Biology and Medicine, University of Freiburg, 79110 Freiburg, Germany; 7 Faculty of Biology, University of Freiburg, 79110 Freiburg, Germany

**Keywords:** herpesvirus, pseudorabies virus, PrV, gD^–^Pass, CRISPR/Cas9 gene editing, sphingomyelin synthase, SMS1, *sgms1*

## Abstract

Herpesviruses are large DNA viruses, which encode up to 300 different proteins including enzymes enabling efficient replication. Nevertheless, they depend on a multitude of host cell proteins for successful propagation. To uncover cellular host factors important for replication of pseudorabies virus (PrV), an alphaherpesvirus of swine, we performed an unbiased genome-wide CRISPR/Cas9 forward screen. To this end, a porcine CRISPR-knockout sgRNA library (SsCRISPRko.v1) targeting 20,598 genes was generated and used to transduce porcine kidney cells. Cells were then infected with either wildtype PrV (PrV-Ka) or a PrV mutant (PrV-gD^–^Pass) lacking the receptor-binding protein gD, which regained infectivity after serial passaging in cell culture. While no cells survived infection with PrV-Ka, resistant cell colonies were observed after infection with PrV-gD^–^Pass. In these cells, sphingomyelin synthase 1 (SMS1) was identified as the top hit candidate. Infection efficiency was reduced by up to 90% for PrV-gD^–^Pass in rabbit RK13-sgms1_KO_ cells compared to wildtype cells accompanied by lower viral progeny titers. Exogenous expression of SMS1 partly reverted the entry defect of PrV-gD^–^Pass. In contrast, infectivity of PrV-Ka was reduced by 50% on the knockout cells, which could not be restored by exogenous expression of SMS1. These data suggest that SMS1 plays a pivotal role for PrV infection, when the gD-mediated entry pathway is blocked.

## 1. Introduction

Many alphaherpesviruses are characterized by a broad host range in vitro and in vivo. This is particularly true for the alphaherpesvirus pseudorabies virus (PrV; Suid alphaherpesvirus 1), causing the notifiable Aujeszky’s disease. Despite the broad host range, pigs, especially domestic pigs and wild boar, are the only natural host and reservoir of PrV [1].

Due to a successful vaccination strategy, domestic pig populations in Western Europe, North America and New Zealand are considered free of PrV. However, Aujeszky’s disease is still endemic in areas with dense pig populations (e.g., (south-)eastern Europe, Latin America, Africa or Asia) causing significant economic losses. Wild boar populations can serve as reservoir for PrV, which includes vertical transmission of the virus to the fetus and leads to infrequent infections of hunting dogs. Thereby the risk for reintroduction of PrV into free regions persists.

PrV can not only infect a wide variety of different cells and tissues in culture, but also a vast number of mammalian species, often with fatal outcome [2,3] indicating that the virus uses ubiquitous cellular proteins for entry and propagation [4]. However, which cellular proteins are essential for virus propagation and their functional role is still not completely understood.

Efficient attachment and entry of PrV requires the concerted action of five different viral glycoproteins. Glycoprotein (g)C mediates primary attachment of the virus particle to the cell surface by binding to heparan sulfate proteoglycans [5,6]. Neither gC nor heparan sulfate, however, are essential for infection and entry is only slightly impaired in the absence of either [5,6,7,8]. For entry, the viral envelope has to fuse with the plasma membrane or with the endosomal membrane after endocytosis [9,10,11,12]. This process is dependent on the coordinated activity of gD, the heterodimeric gH/gL complex and gB [10]. PrV gD binds to a variety of cellular surface proteins including nectin-1 and nectin-2 bringing the viral and cellular membrane in close contact [10,13,14]. Binding to these cellular receptors is thought to induce a conformational change in gD, which then triggers the conserved membrane fusion machinery via the gH/gL complex. This complex activates the actual fusogen gB allowing membrane fusion followed by release of the nucleocapsid and adhering tegument into the cytoplasm [9,10].

While gD, gH/gL and gB are essential for virus entry, only gB and gH are absolutely required for direct cell-to-cell transmission of PrV [15,16]. In the absence of gD or, to a lesser extent, gL, PrV is still able to spread to neighboring cells [15,16,17,18,19]. This phenotype was repeatedly used for “virus evolution in cell culture” by serially passaging mutants lacking gL or gD in eukaryotic cells. This approach resulted in revertants, which gained infectivity in the absence of gL (PrV-ΔgLPass [19,20]) or gD (PrV-gD^–^Pass [17]). In all revertants, mutations in one or more of the other entry glycoproteins were identified [18,20,21] indicating that the viral fusion machinery is flexible enough to adapt to different conditions. Mutations in gB and gH of PrV-gD^–^Pass, were shown to contribute to gD-independent infectivity [21] but the molecular mechanism remains unidentified. It is speculated that in the absence of gD, receptors specific for the gH/gL complex or for gB may play a dominant role [21]. Although HSV-1 gH can bind to different integrins on the cell surface [22,23], it is unclear whether PrV gH/gL and/or gB are able to bind to cellular surface components acting as virus receptors.

After entry, the nucleocapsid and adhering tegument is transported towards the nucleus along microtubules using cellular motor proteins. The viral genome is injected into the nucleus through the nuclear pores. In the nucleus, transcription and DNA replication take place. Capsid proteins are imported into the nucleus using the canonical nuclear import machinery. In the nucleus, they assemble autocatalytically into icosahedral capsids. Progeny capsids in the nucleus are filled with the viral genome and are then released into the cytoplasm by budding through the nuclear membranes depending on yet unknown cellular proteins. In the cytoplasm nucleocapsids mature by addition of tegument proteins and are then incorporated into viral glycoprotein containing vesicles derived from the trans Golgi network or from endosomes. Synthesis and maturation of viral (glyco-)proteins ultimately depends on classical cellular protein synthesis pathways. Mature virus particles are then released after traveling in a transport vesicle using the cellular secretory pathway towards the plasma membrane, where membrane fusion and particle release occur (reviewed in Mettenleiter [24]).

The CRISPR/Cas9 technology offers a straightforward method to study the interaction between host and pathogen in an unbiased high throughput approach [25]. It allows targeting of almost any gene in a genome whose sequence is known, given that at least a specific protospacer adjacent motif (PAM) sequence (“NGG” for Cas9) is present [26]. This allowed the development of genome-wide assays in which every single gene is targeted by one or even multiple single guide RNAs (sgRNAs), usually delivered by a lentiviral vector which results in high transduction efficiencies [27,28,29,30,31]. Several well-characterized lentiviral libraries for human or mouse cells are available [32,33,34,35,36]. These libraries were used to identify host cell factors important for e.g., (bat) influenza virus, flavivirus, norovirus, Schmallenberg virus, human immunodeficiency virus (HIV) and hepatitis C virus [37,38,39,40,41,42,43]. However, sgRNA libraries specific for most of the other mammalian species are still rare.

Here, we generated a porcine sgRNA library (SsCRISPRko.v1), targeting all annotated genes in the porcine genome (*Sus* (*S.*) *Scrofa* 10.2). This library was then used in a genome-wide screen to study host cell factors, which play an important role for PrV infection. This approach resulted in the identification of sphingomyelin synthase 1 (SMS1) as an important entry factor when the canonical gD-mediated entry pathway is blocked.

## 2. Materials and Methods

Cells and viruses. Rabbit kidney cells (RK13, CCLV-Rie 109), human embryonic kidney cells (HEK-293T, CCLV-Rie 1539) and porcine kidney cells (PK15, CCLV-Rie 0005-1) provided by the Collection of Cell Lines in Veterinary Medicine (CCLV) at the Friedrich-Loeffler-Institut, Insel Riems were cultivated in Dulbecco’s modified Eagle´s minimum essential medium supplemented with 10% (RK13) or 5% (HEK-293T, PK15) fetal calf serum (FCS).

PrV wildtype strain Kaplan (PrV-Ka) [44], the ß-galactosidase expressing wildtype-like mutant PrV-1112 [45], and PrV-gD^–^Pass [21] were propagated in RK13 cells. Lentiviruses used in this study were generated as previously described [32,34]. Briefly, lentiviruses were produced in HEK-293T cultured in 10 cm dishes by co-transfection of the respective lentiviral vector plasmids (10 µg) and packaging plasmids psPAX (7.5 µg, Addgene #12260) and pMD2.G (5 µg, Addgene #12259) using Lipofectamine 2000 (Invitrogen, Karlsruhe Germany) resulting in pseudotyped, replication-deficient particles.

Generation of a porcine CRISPR library. A library of sgRNAs for a genome-wide CRISPR knockout (KO) of protein-coding genes in the porcine genome was designed using custom scripts in Python 2.7. Designs were based on the genome assembly *S. scrofa* 10.2 [46], using RefSeq gene models from NCBI *S. scrofa* Annotation Release 105. Predicted genes with no published gene symbol or identified orthologues, with a coding sequence of less than 5000 base pairs were excluded. For each gene, the transcript variant conforming most closely to consensus, as defined by the highest average frequency of exon occurrence across all recognized transcript variants, was selected as the basis for the guide design. First, candidate guides were identified based on the presence of an “NGG” (N: any base; G: guanosine) PAM on either strand giving rise to a predicted Cas9 cut site within an exon, at least 2 nucleotides (nt) from the intron-exon junction. Candidates were rejected if (i) more than one-third of the 30 nt sequence (20 nt protospacer with 4 additional 5′ nt and 6 additional 3′ nt including the PAM sequence) consisted of repetitive or low-complexity elements, (ii) there is more than one ambiguous base, or (iii) the protospacer contained a BsmbI restriction site. Potential cutting efficiency (“on-target score”) was estimated using the Doench Rule Set 2 model in Python package scikit-learn version 0.16.1. [34]. To screen for off-target matches, candidate guide sequences (with appended PAM sequence) were aligned to the genome using BLAT [47], and the likelihood of an off-target match was estimated using a previously published Cutting Frequency Determination (CFD) score [34]. Four guides were chosen for each gene, but if insufficient candidates were available, guides exact matches were permitted, for example to allow targeting of genes with multiple paralogues. Corresponding oligonucleotides were generated by solid phase dense array synthesis by the Broad Institute.

Prior to library synthesis, the suitability of the design was tested using sgRNAs targeting two host genes known to be involved in influenza virus replication [40,48,49]. The sialic acid transporter SLC35A1 was targeted by two sgRNAs, while in addition the transcription factor interferon regulatory factor 7 (IRF7) was targeted by one sgRNA (Table 1). The sgRNAs were cloned into the pLentiCRISPRv2 backbone (Addgene #52961, containing the Cas9 gene of *S. pyogenes*) using the BsmBI restriction overhangs and Lentiviral vectors were generated as described above.

For validation of individual guide cutting efficiency, PK15 cells were transduced with each of the lentiviral vectors as described in the following section and selected with 1.25 µg/μL puromycin for 10 days. To confirm efficient genome editing, genomic DNA (gDNA) was extracted using the DNeasy kit (Qiagen, Hilden, Germany) according to manufacturer’s instructions. The region of gDNA, approximately 300 bp either side of the cut site, was then amplified by PCR using primers given in Table 1. The PCR product was sequenced using Sanger sequencing and the resulting chromatogram was analyzed for cutting efficiency using the TIDE website (http://shinyapps.datacurators.nl/tide/, 14 June 2017).

After the successful preliminary test, the library consisting of 83,381 specific sgRNA and in addition 1000 non-targeting controls was synthesized. Following synthesis, the oligonucleotides were pooled and amplified using non-variable primers that introduce BsmBI restriction sites. The amplified products were then size fractionated by electrophoresis, purified and shotgun cloned into plentiCRISPRv2 (Addgene #52961). To maintain sgRNA representation, multiple repeated cloning reactions were performed and electroporated into highly competent bacteria, which were plated on 25 cm^2^ agar plates. Colonies were harvested by scraping and DNA was extracted by Maxi Kit (Qiagen, Hilden, Germany). Relative coverage and representation of sgRNAs were confirmed by deep sequencing.

CRISPR screening. Lentiviruses encoding the pooled porcine CRISPR-knockout sgRNA library (SsCRISPRko.v1) were generated as described [32,34]. SsCRISPRko.v1 contains 83,381 sgRNAs targeting 20,598 porcine genes and 1000 non-targeting control sgRNAs. Forty million PK15 cells (8 × 20 cm dishes with each 5 × 10^6^ cells) were transduced with the lentiviral sgRNA library at a multiplicity of transduction (MOT) of 0.3 in medium containing 10 µg/mL polybrene [50]. The screening was performed in medium supplemented with penicillin-streptomycin (100 U/mL, Thermo Fisher Scientific, Dreieich, Germany). Two days after transduction, puromycin (1.25 µg/mL) was added and transduced cells were selected and split twice in ten to 14 days. The screening for virus resistant cells started with approx. 3 × 10^8^ cells (30 × 20 cm dishes with 1 × 10^7^ cells each) providing a coverage of >500 cells per sgRNA as recommended [35]. 2 × 10^7^ cells were harvested as “control” and stored pelletized at −20 °C until further analysis.

The remaining cells were infected with PrV (PrV-Ka or PrV-gD^–^Pass) at a multiplicity of infection (MOI) of 0.5. The medium was changed four days after infection and then every week, containing 20% conditioned medium from uninfected and untreated PK15 cells. After approx. ten to 16 days, cell colonies became detectable. These cell colonies were harvested and pooled. Half of the sample was stored at −20 °C and the other half was seeded into 20 cm dishes and re-infected as described above. The survivors of the second infection were harvested approx. 28 days after the first infection, pelletized and stored at −20 °C until further analysis.

Cells were thawed, lysed in sarkosyl buffer (75 mM Tris-HCl, pH 8.3; 25 mM EDTA, pH 8.0; 3% N-lauroylsarcosine in A. dest) for 20 min at 65 °C, treated with RNase A (500 µg/mL, Serva, Heidelberg, Germany) for 30 min at 37 °C and subsequently incubated with Pronase (2 mg/mL, Roche, Mannheim, Germany) at 45 °C overnight. The gDNA was extracted using a standard phenol-chloroform extraction protocol, precipitated with ethanol and dissolved in 10 mM Tris-HCl buffer (pH 8.5).

**Figure 1:**
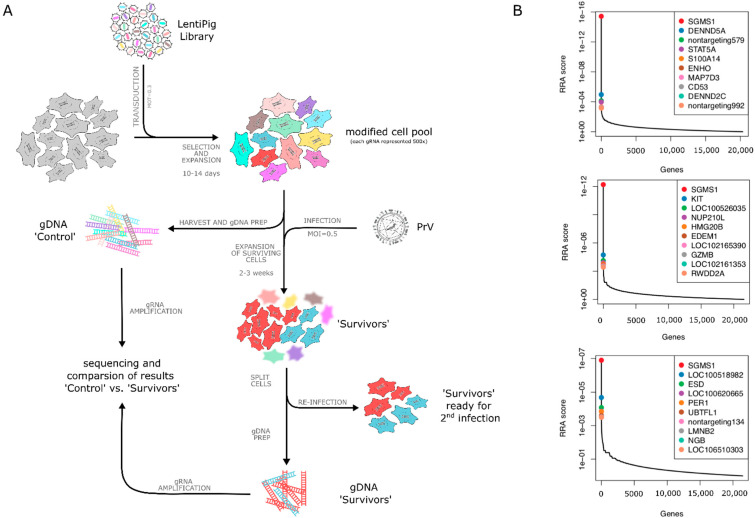
Identification of sphingomyelin synthase 1 (SMS1, *sgms1*) as an entry factor for PrV-gD^–^Pass. (**A**) Schematic overview of the genome-wide CRISPR/Cas9 screening protocol. PK15 cells were transduced with lentiviruses encoding the porcine CRISPR knockout library (SsCRISPRko.v1) at a multiplicity of transduction (MOT) of 0.3. Two days post transduction, cells were selected using puromycin for 10 to 14 days and subsequently infected with PrV at a multiplicity of infection (MOI) of 0.5. Surviving cells were split in half and were either reinfected or harvested to isolate genomic DNA for subsequent analysis by sequencing. (**B**) Genes enriched in the surviving cell pool after infection with PrV-gD^–^Pass were ranked by the robust rank aggregation (RRA) scores, displaying a very strong selection of sgRNAs targeting the *sgms*1 gene in all three independent assays.

The sequencing library was generated by PCR using the different gDNAs as template. For each sample four reactions with each 50 µL PCR reaction mix containing ExTaq DNA polymerase (Clontech, Saint-Germain-en-Laye, France), ExTaq buffer, Mg^2+^, dNTPs, P5-barcode and P7 reverse primers (Table 2) and 5 µg of gDNA were prepared according to manufacturer’s specifications. For each sample, a different P5 barcode primer was used. The PCR was performed as follows: 95 °C for 1 min, 28 cycles of 95 °C for 30 s, 53 °C for 30 s, 72 °C for 1 min with a final amplification at 72 °C for 10 min. After amplification, the four PCR reactions per sample were pooled, the 354 bp fragment was gel purified using a gel extraction kit (Zymo Research, Freiburg, Germany) and eluted in nuclease-free water. Isolation of gDNA, PCR, as well as gel extraction were performed in parallel with the corresponding controls for each set of samples to minimize any bias. The screening procedure is illustrated in Figure 1.

Samples were sequenced on an IonTorrent Ion S5^TM^ XL System (Invitrogen, Dreieich, Germany). The sequencing data were analyzed on the Galaxy web platform (usegalaxy.eu) [48]. The sequencing reads were trimmed to 25 nt sequences with Cutadapt (Galaxy Version 1.16.5) using the 5′ front adapter sequence (TTGTGGAAAGGACGAAACACCG) [49]. The trimmed reads were mapped to the original library file (Supplementary Table S1) analyzing the quality of the sequencing run using the MAGeCK count tool with default settings (Galaxy version 0.5.8.4) [51]. To compare the sequencing results of two different conditions (“Control” vs. “Survivor”) we used the robust rank aggregation (RRA) method of the MAGeCK test tool (Galaxy version 0.5.8.1) [51].

To further evaluate the SsCRISPRko.v1 library performance, an essential gene analysis was performed on the sequencing results from the control cells, representing the modified cell pool after selection and expansion. First, enrichment or depletion of sgRNAs in the control cells relative to the sequenced input plasmid pool was assessed using MAGeCK as above. Target genes were subsequently ranked according to fold change sgRNA enrichment in the cells versus the plasmid stock. Enrichment of essential genes, as defined using previously published reference lists of essential genes [52,53], in gene rankings was evaluated by gene set enrichment analysis in the MAGeCK implementation of GSEA (Galaxy version 0.5.8 [51]), using 10^6^ permutations for *p*-value estimation. The lists for this analysis consisted of: (i) a longer list of 1580 essential genes identified by CRISPR/Cas9 fitness screening in five human cell lines [52], of which 1308 could be matched to unique porcine genes used in the porcine screen or (ii) a refined list of “core” essential genes [53], derived from the previously mentioned long list by network analysis. This included 47 genes matched to unique porcine gene names. We visualized the correlation between the gene expression values (LogFC) as a violin plot. Violin plots were generated in Python 3.7, using package matplotlib v3.1.0.

Generation of RK13-sgms1_KO_ cells. For generation of RK13 knockout (KO) cells four sgRNAs were designed targeting the third exon of sphingomyelin synthase 1 (SMS1; *sgms1*, ENSOCUG00000010965.4) as predicted in the rabbit genome OryCun2.0 (*Oryctolagus cuniculus*, ensemble.org [54]) with the help of the corresponding tool in Geneious software 11.1.5 (https://www.geneious.com, 1 Decmeber 2020). The four gRNAs with the highest score and the lowest probability for off-target effects were selected (Table 3). sgRNAs were ordered as unmodified DNA oligonucleotides (MWG Eurofins, Ebersberg, Germany) with BbsI restriction overhang, hybridized and inserted into the BbsI-digested vector pX330-NeoR as previously described [55,56]. Correct cloning was verified by sequencing with primer HU6-F (ATAATTTCTTGGGTAGTTTGCAG). RK13-sgms1_KO_ cell lines were generated by co-transfection of all four sgRNA-containing pX330-NeoR plasmids (1.5 µg per plasmid) into cells using calcium phosphate-co-precipitation [57].

Two days after transfection in 6 well dishes, cells were transferred to 10 cm plates (Corning, Sigma-Aldrich, Darmstadt, Germany) and selected in medium containing 500 µg/mL G418 (Invitrogen, Karlsruhe, Germany). Ten to 14 days after transfection cell colonies were picked by aspiration and tested for a biallelic KO by sequencing after PCR amplification of the targeted gene sequence using the control primers (Table 3) as described previously [56]. One cell clone, designated as RK13-sgms1_KO_, which contained two deletions in exon 3, was further analyzed.

Generation of SMS1 reconstituted RK13 cell line. RNA from parental RK13 cells was isolated (RNeasy Mini Kit, Qiagen, Hilden, Germany), reverse transcribed using Oligo(dT) primers (RevertAid Kit, Thermo Fisher, Dreieich, Germany) and the *sgms1* cDNA was amplified with specific primers (Table 4) using Phusion High-Fidelity DNA Polymerase (NEB, Frankfurt am Main, Germany) as recommended by the manufacturer. The PCR product was cloned into pLV-X-Puro (Clontech #PT4002-5) using XhoI and BamHI restriction sites added with the primer sequences. Correct PCR and cloning were verified by sequencing. Lentiviruses encoding the *sgms1* gene (sgms1^+^) or empty pLV-X-Puro (+X) were generated in HEK-293T cells as described above [32,34]. RK13-sgms1_KO_ cells were transduced and cells were selected with 1 µg/mL puromycin. SMS1 expression was confirmed by immunoblotting.

Immunoblotting. Cells were seeded in 24 well culture dishes and harvested two days post seeding by scraping into the medium, pelletized, washed twice with 1× phosphate-buffered saline (PBS) and lysed in SDS-containing sample buffer (0.13 M Tris-HCl, pH 6.8; 4% SDS; 20% glycerin; 0.01% bromophenol blue; 10% 2-mercaptoethanol). Proteins were separated in SDS 10% polyacrylamide gels and after transfer to nitrocellulose membranes, blots were probed with an SMS1 specific antibody (1:1000, HPA045191, Sigma Aldrich, Darmstadt, Germany) and an anti-α-tubulin antibody (1:10,000, Sigma-Aldrich, T5168) as loading control. After incubation with secondary peroxidase-labelled antibodies and substrate (Clarity ECL western Blot substrate, Bio-Rad, Feldkirchen, Germany), chemiluminescence was recorded in a Bio-Rad Versa Doc imager.

Cell Viability Assay. Cell viability was analyzed using the PrestoBlue™ Reagent (Thermo Scientific, Dreieich, Germany), a resazurin-based metabolic assay. For this, 1 × 10^4^ cells were seeded in 90 µL volume in a black 96 well plate with a flat and clear bottom (Corning, Sigma-Aldrich, Darmstadt, Germany). At 24, 48 and 72 h after seeding 10 µL PrestoBlue^TM^ Reagent was added to the cell culture supernatant and mixed. The samples were incubated for 30 min at 37 °C. For each time point, six replicates were used including six wells containing medium for background estimation. Before bottom-read measuring of fluorescence in a Tecan Reader at Ex_560 nm_/Em_590 nm_, the plate was agitated for 5 sec. Multiple reads per well (3 × 3) were performed using the i-control™ microtiter reader software. Blank-reduced raw data (fluorescence intensities) are given, while the statistics were done with standardized values. For standardization, the following formula was used: (measured value—mean)/standard deviation.

Oil Red O Staining. The intracellular lipid distribution was visualized by Oil Red O staining. For this, 5 × 10^5^ cells were seeded on coverslips in a 6 well plate, incubated for 24 h and then fixed with 4% paraformaldehyde in 1× PBS for 15 min. Fixed cells were washed three times with 50 mM NH_4_Cl in 1× PBS and then incubated for 30 min in the same buffer to quench the free aldehyde groups. A 0.3% Oil Red O (Serva, Heidelberg, Germany) stock solution was prepared in 100% 2-propanol. The Oil Red O stock solution was diluted in A. dest (60% Oil Red O Stock) and filtered through a 0.45 µm syringe filter unit. After two washing steps with A. dest, the cells were incubated for 30 min in diluted Oil Red O staining solution. Subsequently, cells were washed five times in A. dest, nuclei were counterstained for 10 min with Hematoxylin solution (Medite, Burgdorf, Germany) and cells were mounted in a drop of aqueous mounting medium (Aquatex, Merck, Darmstadt, Germany). The cells were imaged using the Aperio ScanScope CS system (Leica, Wetzlar, Germany). Scale bars indicate 50 µm.

Membrane staining. For membrane staining, 5 × 10^5^ cells were seeded on coverslips in a 6 well plate and treated for 24 h with CellBrite^TM^ (Neuro DiO-based, 1:600 in cell culture medium, Hölzel Diagnostika, Köln, Germany) before fixation with 4% paraformaldehyde in 1× PBS for 15 min. Fixed cells were washed three times with 50 mM NH_4_Cl in 1× PBS and then incubated for 30 min in the same buffer. Membranes were directly visualized via the autofluorescence of the staining dye. The nuclei were counterstained with 300 mM DAPI for 5 min and cells were mounted in a drop of Kaiser’s glycerol gelatin (Merck, Darmstadt, Germany). Samples were analyzed using a confocal laser scanning microscope (Leica DMI 6000 TCS SP5, 63× oil-immersion objective, NA = 1.4; Leica, Wetzlar, Germany). The different cell lines were processed in parallel and all images were acquired with identical parameters (laser excitation, detector settings, and zoom factor). Representative single images were processed using the Fiji software version 1.52 [58,59]. Scale bars indicate 10 µm.

Quantification of the cellular fluorescence was performed using Fiji software as previously described [60]. The integrated density (IntDen) of 25 cells was measured from five independent fields of view (5 cells per view, 80 × 80 µm) and in each view, two regions without fluorescence were measured for background reduction. The corrected total cell fluorescence (CTCF) was calculated using the following formula: CTCF = IntDen – (area of selected cell × mean fluorescence of background).

In vitro replication studies. To test for PrV propagation, RK13 and RK13-sgms1_KO_ cells were seeded with 2 × 10^5^ cells per 24 well and subsequently infected with wildtype-like PrV-1112 or PrV-gD^–^Pass at an MOI of 5. Cells and supernatants were harvested at different time points after infection (0 h, 4 h, 8 h, 12 h, 24 h and 30 h p.i.) and stored at –80 °C. To determine the infectious virus titer, samples were thawed and cell debris was removed by centrifugation (2 min, 14,000× *g*). The supernatant was serially diluted (10^−1^ to 10^−6^) and used to infect RK13 cells in 48 well culture plates. After incubation for 1 h, the inoculum was replaced by a semi-solid medium allowing only direct cell-to cell spread of the virus. Cells were fixed after 2 days with formaldehyde and stained with crystal violet. Virus plaques were counted in at least two wells and mean values were calculated as plaque forming units per milliliter (pfu/mL). Shown are mean values of three independent experiments with corresponding standard deviations.

Determination of efficiency of plating. To check for efficiency of plating, cells were seeded at 3 × 10^5^ cells per 12 well and subsequently infected with PrV-1112 or PrV-gD^–^Pass each with 200 pfu per well. After incubation for 1 h, the inoculum was replaced by a semi-solid medium. Cells were fixed after 2 days and stained with 5-bromo-4-chloro-3-indolyl-ß-D-galactopyranoside (X-Gal). The efficiency of plating was determined by counting the number of plaques in comparison to the RK13 control, which was normalized to 100%.

Statistics. For each assay at least three independent experiments were performed. The statistical significance of the data was determined by statistical tests using GraphPad Prism version 8.1.0. A *p*-value ≤ 0.05 was considered significant and is presented in the figures in form of asterisks (* ≤0.05, ** ≤0.01, **** ≤0.0001).

## 3. Results

Design of the porcine CRISPR library. A broadly applicable porcine sgRNA library for genome-wide knockout screens was designed based on the genome assembly of *S. scrofa* 10.2 [46]. For each gene, preferentially four specific 20 nt-sgRNA sequences were chosen that met the following criteria: (i) no exact off-target matches elsewhere in the genome, (ii) on-target score >0.2, maximum CFD score for an off-target match < 0.2, (iii) separated from a higher ranked guide by at least 5% of the coding sequence and (iv) located within the first 5–65% of the coding sequence. Where more than four candidate guides met these criteria, on-target scores (rounded to one decimal place) and off-target scores were used to rank guides further.

A total of 20,598 genes were targetable, with four guides each for 20,582 of them. The final library designated as SsCRISPRko.v1 contains 83,381 sgRNAs and 1000 non-targeting controls (Supplementary Table S1). The sgRNA design was validated by testing specific sgRNAs against host genes previously described to be important for influenza virus infection [40,48,49]. For this, PK15 cells were transduced with lentiviral vectors targeting SLC35A1 (two sgRNAs) or IRF7 (one sgRNA) (Table 1). Following puromycin selection and gDNA isolation, the genomic region surrounding the cutting site was amplified from both transduced and non-transduced cells and sequences were analyzed using the web tool TIDE. This web tool compares the sequencing trace around the CRISPR cut site in pools of edited versus unedited DNA to estimate the spectrum and frequency of small edits. This is achieved by analyzing the shifts in sequence signal from small insertions and deletions (InDels) around the cut site [61].

From the cutting site onwards, the sequence from edited cells consisted of a mixture of signals derived from unedited DNA and from sequences shifted by a different number of nucleotides due to InDels [61]. Results shown in Supplementary Figure S1 illustrate the cutting efficiency with InDels generated from each guide thereby validating the sgRNA design and efficiency.

SsCRISPRko.v1 library performance. The performance of the porcine library was analyzed by sequencing the plasmid stock and the control cells after selection and expansion. In the porcine library plasmid stock > 99.95% of the sgRNAs were detected in three independent sequencing replicates with a mean zero count of 39. The sgRNA distribution of the library is persuasive, measured by a mean GiniIndex of 0.06343 (*n* = 3) assessed with MAGeCK Count. This shows that the synthesized library has a great overlap with the computationally designed library, without major losses during cloning and amplification.

CRISPR knockout of genes, which are essential for cell survival, should result in the depletion of the corresponding sgRNA sequences in the gDNA derived from the control cell pool after selection and expansion. To further evaluate the performance of the library, an essential gene drop-out analysis was done by comparing the read counts from the control cell pool and the input plasmid set. Genes were ranked by fold change (FC) in control cells vs. plasmid stock and compared in an enrichment analysis using two reference lists of essential genes [52,53]. sgRNAs targeting essential genes were significantly depleted in the transduced control cells compared to the input plasmid stock in all three replicates as shown by enrichment in higher-ranked genes, ranked in ascending order of fold change, in gene set enrichment analysis (GSEA, implemented in MAGeCK GSEA, version 0.5.8). A representative violin plot of one screen, as well as the statistical details of all three screens are given in Supplementary Figure S2. Depletion of sgRNAs targeting essential genes compared to the input pool confirms successful targeting by the porcine CRISPR/Cas9 library and negative selection in the initial phase of cell culture prior to infection, indicating efficient library performance.

Identification of SMS1 as entry factor for PrV. Before starting the genome-wide CRISPR screen, the knockout efficiency of Cas9 in PK15 cells was tested. Cells were transduced with lentiCRISPRv2 expressing a sgRNA targeting EGFP (Lenti-CRISPRv2_EGFP-sgRNA_) or with control (Lenti-CRISPRv2_empty_) and then selected with puromycin (1.25 µg/mL) until the non-transduced control cells were dead. After selection and expansion, cells were subsequently transduced with lentivirus expressing EGFP. Knockout efficiency of Cas9 was monitored by reduction of the EGFP signal using flow cytometry 3 days post transduction. We observed more than 97% reduction of EGFP positive cells compared to lentiCRISPRv2_empty_ transduced cells (Supplementary Figure S3). This indicated that PK15 cells are suitable tools for the genome-wide CRISPR/Cas9 screen.

The workflow for the genome-wide screen is outlined in Figure 1A. The porcine lentiviral library was used to transduce PK15 cells at an MOT of 0.3, to enhance the probability that every cell is transduced with only one sgRNA. The transduced cells were selected with puromycin and expanded for ten to 14 days to achieve at least 500-fold coverage for each sgRNA as recommended [35]. From this cell pool, 2 × 10^7^ cells were harvested as control and stored for further analysis. The remaining cell pool was then infected with either PrV-Ka or PrV-gD^–^Pass at an MOI of 0.5.

Interestingly, no cells survived the infection with PrV-Ka in repeated assays, even after a prolonged incubation of up to three months indicating that PrV can use a wide panel of different host cell factors for successful infection. Because of this, we tested a mutant, PrV-gD^–^Pass, which lacks the receptor-binding protein gD but regained infectivity after repeated passages in cell culture [17]. Mutations in gB and gH have been shown to be involved in the gD-independent entry pathway but the exact mechanism is yet unknown [21]. Our genome-wide CRISPR/Cas9 approach should shed more light on this mode of infectious entry.

After infection with PrV-gD^–^Pass single cell colonies were visible after 10 days post infection (p.i.). These cell colonies were harvested 14 to 16 days p.i., pooled and one half of the cell pool was saved as ‘survivors #1’ for gDNA isolation and sequencing, while the other half was reinfected with PrV-gD^–^Pass at an MOI of 0.5. ’Survivors #2′ were harvested 12 to 14 days after the second round of infection. The screening was done in three independent assays. For each assay, gDNA of all cell pools (control cells before infection, survivors #1 and #2) was isolated in parallel to avoid bias and used for PCR to amplify the integrated CRISPR regions. PCR products were purified and subjected to Ion Torrent sequencing.

Sequencing data were analyzed using the MAGeCK platform [51] which identified the significantly enriched sgRNAs present in the gDNAs of survivors compared to the non-infected control cells. Based on a robust rank aggregation (RRA) algorithm the absolute top hit candidate, with all four sgRNAs found enriched was identified as sphingomyelin synthase 1 (SMS1, *sgms1*) (Figure 1B), a protein involved in sphingolipid metabolism. The other genes among the top 10 were found with a significantly higher and therefore less stringent RRA score and varied between the different independent assays, clearly separating them from the top hit SMS1.

Generation of a RK13-sgms1_KO_ cell line. Based on results from three independent screenings, we set out to validate the top hit by analyzing the role of SMS1 in our standard cell line, rabbit kidney cells (RK13). Although RK13 cells are not the natural host cells for PrV, they propagate the virus to high titers within a short time, are easy to transfect and no differences in the PrV replication cycle compared to cells of pig origin are evident.

**Figure 2:**
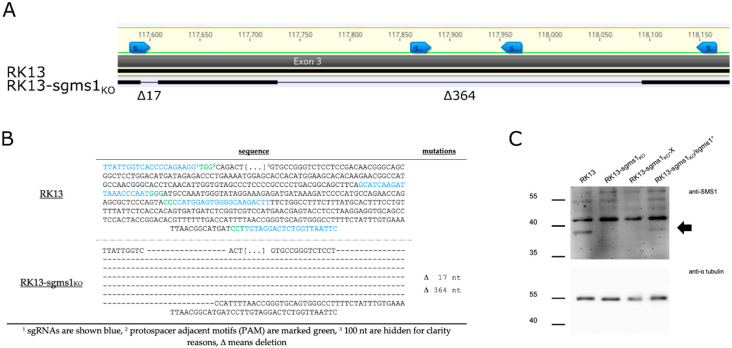
Characterization of RK13-sgms1_KO_ cells. (**A**) Shown is the targeted sequence of exon 3 of the rabbit *sgms1* gene region. The sequence derived from RK13-sgms1_KO_ (lower line) was compared to the parental rabbit sequence (ENSOCUG00000010965.4, upper line). Due to the large deletion (Δ) identical regions between wildtype and knockout are indicated by black bars and the deleted sequence is represented by a thin line. A detailed analysis on base pair level is shown in (**B**). Locations of sgRNAs are marked in blue (**A**,**B**), while the corresponding PAM sequence is marked in green. Deletions are shown by hyphen. (**C**) Lysates of wildtype RK13 and RK13-sgms1_KO_, as well as RK13-sgms1_KO_ cells transduced either with an empty vector (RK13-sgms1_KO_-X) or with a vector expressing the rabbit SMS1 (RK13-sgms1_KO_/sgms1^+^) were probed with a SMS1-specific antiserum. The specific ca. 38 kDa band is marked by an arrow. The α-tubulin monoclonal antibody was used as loading control. Molecular masses of marker proteins (in kDa) are indicated on the left.

*Sgms1* knockout RK13 cell lines were generated by cloning the four sgRNA specific oligonucleotides targeting the rabbit homologue of SMS1 into the Cas9 encoding vector pX330-NeoR [55]. Two of the four sgRNA sequences were identical to those present in the porcine library. All four plasmids were co-transfected into RK13 and cells were selected with 0.5 mg/mL G418. Single G418-resistant cell clones were picked approx. 14 days post transfection and the gDNA was isolated. The targeted *sgms1* gene region was amplified by PCR with specific primers (Table 3), cloned and sequenced as described before [56]. The cell line, which was chosen for further analyses, carried two deletions comprising 17 nt and 364 nt in exon 3 of *sgms1*, removing amino acids 15 to 182 and introducing a frame shift (Figure 2A). Direct sequencing of the PCR product uncovered the modification with no sequence bias, already indicating a biallelic knockout. To further validate this, the PCR product was cloned into the vector pBluescript and the inserts of 10 randomly selected plasmid clones was determined, which is generally accepted for verifying CRISPR-engineered modifications [62]. All cloned PCR products derived from RK13-sgms1_KO_ cells revealed the same deletions further showing that both alleles carry the identical modification (Figure 2B).

Western blot analysis using an SMS1-specific antibody showed absence of the approx. 38 kDa signal in RK13-sgms1_KO_ compared to RK13 cell lysates (Figure 2C) confirming the successful knockout of full-length SMS1 also on protein level. No smaller sized bands were detectable in RK13-sgms1_KO_ cell lysates, but the serum used was raised against the N-terminal 120 amino acids of SMS1. In case translation would start at a downstream codon, the truncated isoforms would not be detected by the specific antiserum. However, the corresponding protein product would lack the conserved sterile alpha motif (SAM) and at least the first of the predicted six transmembrane domains. The approx. 45 kDa signal detectable in all cell lysates is probably due to background staining.

**Figure 3:**
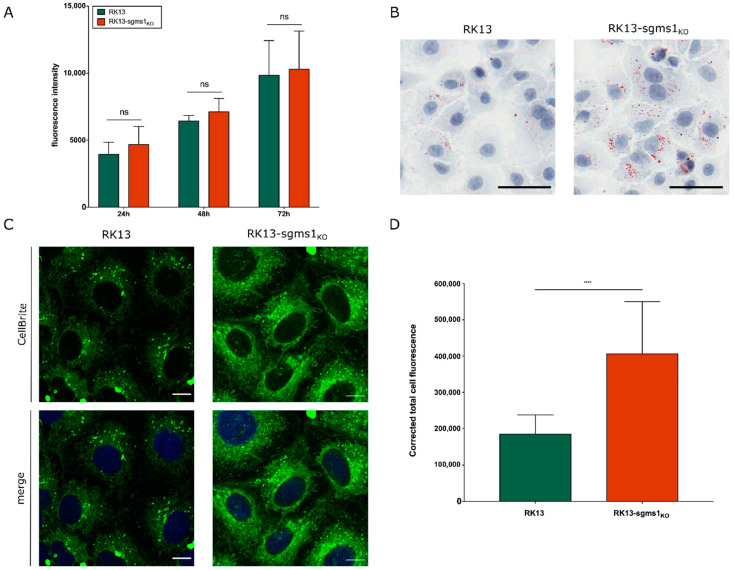
Characterization of RK13-sgms1_KO_ cells. SMS1 knockout cells were compared to parental RK13 cells. Cell viability and propagation was measured at 24, 48 and 72 h post seeding using the PrestoBlue^TM^ reagent (**A**). Shown is the mean of three independent experiments. Differences in the lipid metabolism were detected by Oil Red O staining (red) showing an accumulation of lipids in RK13-sgms1_KO_ cells compared to parental RK13 cells (**B**). Nuclei were counterstained with Hematoxylin and scale bars indicate 50 µm. (**C**) Cytoplasmic membranes were stained with CellBrite^TM^ for 24 h. Nuclei were counterstained with DAPI, and fluorescence was detected with a confocal laser-scanning microscope with constant settings. Scale bars indicate 10 µm. (**D**) Fluorescence was quantified by calculation of the corrected total cell fluorescence. Significant differences were calculated by two-way ANOVA followed by Sidak’s multiple comparison test (**A**, ns: not significant) or by an unpaired *t*-test (**D**, **** ≤0.0001).

Characterization of RK13-sgms1_KO_ cells. The metabolic activity of RK13 and RK13-sgms1_KO_ cells was measured to test whether the SMS1 knockout affects cell proliferation and viability. For this, the resazurin-based PrestoBlue^TM^ reagent was used. RK13 and RK13-sgms1_KO_ cells were seeded in 96 well plates, and cell viability was measured at 24 h, 48 h and 72 h, respectively. No significant differences in fluorescence intensity between the parental and the RK13-sgms1_KO_ cells were found (Figure 3A) indicating that absence of SMS1 does not impair cell viability in presence of FCS.

Since SMS1 plays a crucial role in sphingolipid metabolism [63] and *sgms1* deficiency was described to unbalance sphingolipid levels [64], we tested for an effect in the RK13-sgms1_KO_ cells. To detect the presence of neutral lipids, RK13 and RK13-sgms1_KO_ cells were fixed and stained with Oil Red O. In comparison to native RK13, RK13-sgms1_KO_ cells showed an accumulation of lipid dots in the cytoplasm (Figure 3B) indicating that the lipid metabolism in RK13-sgms1_KO_ cells is altered.

To analyze whether SMS1 knockout results in differences in intracellular membranes we used CellBrite Dye, which labels preferentially cytoplasmic membranes after longer incubation times. Here, we stained the RK13 and RK13-sgms1_KO_ cells for 24 h before imaging by confocal microscopy. Imaging was performed with constant settings for both cell lines. As shown in Figure 3C,D, fluorescence intensity is significantly higher in the SMS1 knockout cells indicating an accumulation of cytoplasmic membranes.

**Figure 4:**
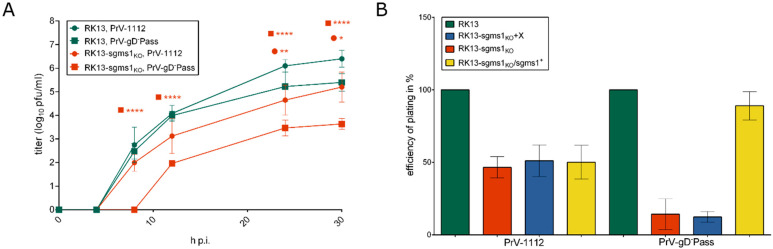
Effect of *sgms1* gene knockout on PrV replication in RK13 cells. (**A**) RK13 and RK13-sgms1_KO_ cell lines were infected with PrV-1112 or PrV-gD^–^Pass at an MOI of 5 and harvested at different times p.i. Progeny virus titers were determined on RK13 cells. Given are mean values as log_10_ plaque forming units (pfu) per ml of three independent experiments with corresponding standard deviations. Asterisks indicate statistically significant differences calculated by two-way ANOVA followed by Sidak’s multiple comparison test compared to the parental RK13 in the same symbol as the corresponding graph (* ≤0.05, ** ≤0.01, **** ≤0.0001). (**B**) For determination of the efficiency of plating (EOP) cells were infected with approx. 200 pfu per 12-well. The EOP was determined by counting plaques two days post infection and the numbers were normalized (in %) to parental RK13. Given are mean values of six independent experiments with corresponding standard deviations.

SMS1 is required for efficient entry of PrV-gD^–^Pass. To analyze the importance of SMS1 on PrV replication, RK13 and RK13-sgms1_KO_ cells were infected with either the wildtype-like PrV-1112 (ßGal^+^) or PrV-gD^–^Pass (gD^–^, ßGal^+^) at an MOI of 5. Cells and supernatant were harvested at different time points p.i. and progeny virus titers were determined on parental RK13 cells. As depicted in Figure 4A, titers for PrV-1112 derived from RK13-sgms1_KO_ cells were approx. 10-fold lower, while titers of PrV-gD^–^Pass were more than 100-fold reduced compared to the parental RK13 cells.

To test whether this effect is congruent with a defect in entry, cells were infected with approx. 200 pfu/well under plaque assay conditions, stained with XGal solution and plaques were counted two days p.i. As shown in Figure 4B, efficiency of plating was approx. 50% reduced for PrV-1112, while only 10–20% of plaques were observed for PrV-gD^–^Pass compared to the parental RK13 cells (set to 100%). This indicates that SMS1 plays a prominent role in entry related pathways of PrV infection.

To test whether the observed effect is indeed due to the defect in SMS1 expression, we reconstituted RK13-sgms1_KO_ cells with SMS1. RNA of RK13 cells was isolated, reverse transcribed and the *sgms1* open reading frame was amplified with specific primers (Table 4). The 1.2 kb PCR product was cloned into pLV-X (pLV-sgms1^+^) and RK13-sgms1_KO_ cells were transduced either with the empty vector (pLV-X) or with pLV-sgms1^+^. Transduced cells were selected in medium containing puromycin. In comparison to cells transduced with the empty control lentivirus, the 38 kDa protein was detectable in the restored RK13-sgms1_KO_/sgms1^+^, albeit with reduced intensity compared to parental RK13 cells (Figure 2B). Since we used the original, unmodified *sgms1* coding sequence, it might be speculated that Cas9 in combination with the sgRNAs is still partially effective and modifying the exogenous gene. Despite this reduced expression, efficiency of plating was increased for PrV-gD^–^Pass to approx. 90% in RK13-sgms1_KO_/sgms1^+^ cells but remained at a 50% level for wildtype-like PrV-1112 (Figure 4B) indicating that SMS1 plays a prominent role when the gD-mediated entry pathway is blocked.

## 4. Discussion

We generated a porcine-specific library, designated as SsCRISPRko.v1 to investigate host factors important for PrV infection. Although the function of most of the viral proteins in the PrV replication cycle is already understood to some detail [65,66] knowledge on host cell factors involved in efficient virus propagation is still lagging. The generated SsCRISPRko.v1 library contains 83,381 sgRNAs and 1000 control sgRNAs targeting a total of 20,598 genes. Sequencing, computational and experimental analyses using sgRNAs specific for host cell factors important for influenza virus infection and the depletion of sgRNAs targeting essential genes in the modified control cell pool confirmed a good performance of our newly designed library.

Recently, in parallel to our study, a genome-scale porcine CRISPR/Cas9 knockout library designated as PigGeCKO, with sgRNAs targeting 17,774 protein-coding genes, 11,053 long non-coding RNAs and 551 micro-RNA was used to identify host factors associated with Japanese encephalitis virus (JEV) replication [67]. While PigGeCKO requires Cas9-expressing cells, our library is cloned in a vector which also carries the genetic information for the Cas9 nuclease, which might render our library more broadly and easily applicable.

In our first screens, we used wildtype PrV-Ka for selection, but no cells survived. The same was true when we infected the cells with the highly attenuated vaccine strain Bartha [68]. This is in line with the perception that PrV is very flexible, can adapt to a number of different settings and can use multiple host cell proteins for successful propagation [4]. In contrast, infection of our porcine CRISPR library modified PK15 cell pool with a virus mutant, which is infectious in the absence of gD, PrV-gD^–^Pass, resulted in infection-resistant cells. Sequence analysis of surviving cells from three independent assays uncovered that all four sgRNAs directed against *sgms1*, which encodes the enzyme sphingomyelin synthase (SMS) 1, were found highly enriched and ranked on the top of the hit list. RRA values clearly separated the *sgms1* hit from the other genes in the top 10 list, which in addition differed between the individual screens and were therefore not further analyzed.

SMSs synthesize sphingomyelin (SM) by transfer of phosphocholine from phosphatidylcholine to ceramide, yielding diacylglycerol as a side product [69]. Sphingolipids, such as SM and ceramides are essential and ubiquitous constituents of membranes and have distinct roles in a wide variety of cellular functions, e.g., in barrier function or the regulation of signaling pathways [63]. Proteins encoded by *sgms1* (SMS1) and *sgms2* (SMS2), which are distinct in their expression pattern and subcellular localization, have been identified with SM synthesis activity in mammalian cells [64]. While SMS1 is located in the lumen of the Golgi apparatus, SMS2 is present mainly in the outer leaflet of the plasma membrane [70]. Both contribute to SM production [71] and are required for SM homeostasis and cell growth. Since SMSs are active at a key point in sphingolipid synthesis, blocking the activity not only influences levels of SM and ceramide, but also of other related sphingolipids like glycosphingolipids (GSL), sphingosine and spingosin-1-phosphate [72,73].

To validate the *sgms1* hit in a different host cell background, we generated a *sgms1*-knockout cell line of our standard rabbit kidney (RK13) cell line. The RK13-sgms1_KO_ cell clone isolated after targeted CRISPR/Cas9 mutagenesis carries two deletions, comprising 17 nucleotides and 364 nucleotides, respectively, affecting codons 15 to 182 of the rabbit homologue and additionally resulting in a frame shift in exon 3. Absence of a specific approx. 38 kDa protein could be demonstrated by western blot analysis using a monospecific antiserum raised against the N-terminal 120 amino acids of the human SMS1 homolog. SMS homologs are found throughout the animal kingdom and constitute multipass membrane proteins with six predicted transmembrane domains, with the N- and the C-terminal domain reaching into the cytosol [69]. Unfortunately, reagents detecting the C-terminal part were not available. However, if a truncated *sgms1* gene product would be expressed in RK13-sgms1_KO_ cells it would lack at least one-third of the N-terminal domain including the sterile alpha motif (SAM) and the first transmembrane domain. Deletion of the SAM domain of human SMS1 resulted in a significant reduction of SM synthesis [74] indicating that even if a truncated SMS1 product is expressed it will most likely be functionally impaired.

Although SMSs play an important role for cell growth and propagation, RK13-sgms1_KO_ cells are viable and exhibited no impairment in metabolic activity and cell division. Viability was measured in the presence of fetal calf serum and SM levels present seem to be sufficient to compensate the defect in SMS activity [64]. To test for a functional effect in our RK13-sgms1_KO_ cell line, we analyzed whether lipid distribution is altered. Both, Oil Red O, which predominantly stains neutral lipids and CellBrite labeling of intracellular membranes, showed a significantly higher presence of lipids in RK13-sgms1_KO_ compared to the parental RK13 cells indicating that the knockout was successful. While we specifically detected all four sgRNAs directed against the *sgms1* gene in our screening, none against SMS2 were found to be enriched after infection with PrV-gD^–^Pass.

Infection of the RK13-sgms1_KO_ cell line demonstrated the importance of SMS1 for entry of PrV-gD^–^Pass. Only approx. 10% to 20% of plaques were formed on RK13-sgms1_KO_ compared to RK13 cells. This defect could be restored by heterologous expression of wildtype SMS1 after lentivirus transduction validating that the observed defect is indeed due to inactivation of the cellular *sgms1* gene. Although the number of plaques was also reduced for wildtype-like PrV-1112, restoration of SMS1 expression could not rescue this defect indicating that either the expression level of SMS1 was too low to compensate for the defect in PrV-1112 or that the reduction is not related to the impaired SMS1 function. This needs further investigation. Interestingly, PrV entry was inhibited by 54% in cells treated with sphingomyelinase [75]. Due to their distinct expression pattern and subcellular localization [64], we do not expect a SMS2 knockout or a SMS1/SMS2 double knockout to yield comparable or additive inhibitory effects on PrV entry pathways.

Sphingomyelin and other sphingolipids have previously been described to have an impact on attachment and entry of different viruses, e.g., JEV, HIV and Ebola virus [76,77,78]. Interestingly, an altered turnover of SM and GSL was observed in PrV and HSV infected cells [75,79,80,81,82]. In addition, *sgms1* was found in a CRISPR-based screen analyzing bovine cells in BoHV-1 infection ([83], bioRxiv preprint). However, the study did not analyze SMS1 in more detail.

It has been proposed that lipid rafts, which are highly enriched in cholesterol and sphingolipids, are structured platforms that carry specific proteins and are preferentially used by viruses in various steps during replication [84,85,86]. SMS1 deficiency was shown to significantly decrease SM levels in plasma membranes [73,87]. Many viruses e.g., influenza virus or vesicular stomatitis virus [88,89] were described to induce and use lipid rafts as docking point for subsequent invagination from the plasma membrane in intracellular clathrin-coated vesicles [90,91]. Alphaherpesvirus receptor proteins like HVEM or nectin-1 are not associated with lipid rafts but HSV-1 gB was described to rapidly mobilize lipid rafts to serve as a platform for entry [92]. Although the pathway of PrV-gD^–^Pass entry has not yet been elucidated, the association of gB with lipid rafts on the cell surface might play a crucial role for gD-independent entry.

Although loss-of-function CRISPR/Cas9 screenings are powerful tools to identify cellular factors relevant for virus replication, this approach faces limitations, especially if factors are essential for host cell survival or if multiple factors exist—as this appears to be the case for PrV and probably also for other (alpha)herpesviruses. However, the data we present highlight the power of CRISPR screens using our newly generated library SsCRISPRko.v1 allowing further unraveling of pig–pathogen interactions, an important livestock species. Thus, our porcine library SsCRISPRko.v1 and the recently published PigGeCKO library [67] are valuable tools for CRISPR screens to identify cellular factors relevant for the replication of a multitude of porcine pathogens including swine influenza virus (SIV), porcine epidemic diarrhea virus (PEDV), porcine circoviruses (PCV) or the emerging African swine fever virus (ASFV).

## Figures and Tables

**Table 1:** sgRNA sequences and control primers used to confirm cutting efficiencies.

Name	Function	Sequence (5′ to 3′)
SLC35A1_sgRNA1	sgRNA sequence	GTATGCTGTTCAGAACAACA
SLC35A1_sgRNA2	sgRNA sequence	GGTATAAGCTGCAGCCACCA
IRF7_sgRNA1	sgRNA sequence	GGTGCCGAAGTCGAAGATGG
SLC35A1_ctrl_sgRNA1_Fwd	primer	AGGATGCATTGCTGGTATGTTT
SLC35A1_ctrl_sgRNA1_Rev	primer	AAAGCAGTGCAGGGAATCTTCA
SLC35A1_ctrl_sgRNA2_Fwd	primer	AGCATTTTGAGGTACAATGTTCA
SLC35A1_ ctrl_sgRNA2_Rev	primer	CTCTCAGCATCCTTGGCCTC
IRF7_ ctrl_sgRNA1_Fwd	primer	TACAAAGGTCGAACGGTGCT
IRF7_ ctrl_sgRNA1_Rev	primer	GCTCCAACTGCGGGTAGG

**Table 2:** Oligonucleotide sequences for IonTorrent Sequencing.

Name	Sequence (5′ to 3′)
ITA2fwd_ID85_P5both	CCATCTCATCCCTGCGTGTCTCCGACTCAGCCAGCCTCAACGATTTGTGGAAAGGACGAAACACCG
ITA2fwd_ID86_P5both	CCATCTCATCCCTGCGTGTCTCCGACTCAGCTTGGTTATTCGATTTGTGGAAAGGACGAAACACCG
ITA2fwd_ID87_P5both	CCATCTCATCCCTGCGTGTCTCCGACTCAGTTGGCTGGACGATTTGTGGAAAGGACGAAACACCG
ITA2fwd_ID88_P5both	CCATCTCATCCCTGCGTGTCTCCGACTCAGCCGAACACTTCGATTTGTGGAAAGGACGAAACACCG
ITA2fwd_ID89_P5both	CCATCTCATCCCTGCGTGTCTCCGACTCAGTCCTGAATCTCGATTTGTGGAAAGGACGAAACACCG
ITA2fwd_ID90_P5both	CCATCTCATCCCTGCGTGTCTCCGACTCAGCTAACCACGGCGATTTGTGGAAAGGACGAAACACCG
ITA2fwd_ID91_P5both	CCATCTCATCCCTGCGTGTCTCCGACTCAGCGGAAGGATGCGATTTGTGGAAAGGACGAAACACCG
ITA2fwd_ID92_P5both	CCATCTCATCCCTGCGTGTCTCCGACTCAGCTAGGAACCGCGATGATTTGTGGAAAGGACGAAACACCG
ITA2fwd_ID93_P5both	CCATCTCATCCCTGCGTGTCTCCGACTCAGCTTGTCCAATCGATTTGTGGAAAGGACGAAACACCG
ITA2fwd_ID94_P5both	CCATCTCATCCCTGCGTGTCTCCGACTCAGTCCGACAAGCGATTTGTGGAAAGGACGAAACACCG
ITA2rev_IDxx_P7leCrV2	CCTCTCTATGGGCAGTCGGTGATCCAATTCCCACTCCTTTCAAGACCT

**Table 3:** Oligonucleotide sequences. Compatible 5′ overhangs for restriction enzyme BbsI used for cloning are underlined.

Name	Sequence (5′ to 3′)
sgms1_ctrl_seq_Fwd	GCGAGTCCCACCATCTTGAT
sgms1_ctrl_seq_Rev	GCTACCCAGCCAGTCATAGG
sgms 1_sgRNA#1_Fwd	CACCTTATTGGTCACCCCAGAAGG
sgms 1_sgRNA#1_Rev	AAACCCTTCTGGGGTGACCAATAA
sgms 1_sgRNA#2_Fwd	CACCGCATCAAGATTAAACCCAAT
sgms 1_sgRNA#2_Rev	AAACATTGGGTTTAATCTTGATGC
sgms 1_sgRNA#3_Fwd	CACCTTCAGAACGGGGTGAGGTAC
sgms 1_sgRNA#3_Rev	AAACGTACCTCACCCCGTTCTGAA
sgms 1_sgRNA#4_Fwd	CACCCTTAATTGGTCTCAGGATGT
sgms1_sgRNA#4_Rev	AAACACATCCTGAGACCAATTAAG

**Table 4:** *sgms1* cDNA Primers. Restriction enzyme sites XhoI (Fwd) and BamHI (Rev) used for cloning are underlined.

Name	Sequence (5′ to 3′)
sgms1-cDNA_Fwd	GATCCTCGAGATGTCGTCTGCCAGTACAATG
sgms1-cDNA_Rev	GATCGGATCCTTACGTGTCGTTCACCAGTC

## Data Availability

The data presented in this study and not included in the supplement are available on request from the corresponding author.

## Supplementary Materials

The following are available online at https://www.mdpi.com/article/10.3390/v13081574/s1, Table S1: SsCRISPRko.v1 library, Figure S1: TIDE analysis of gene regions, Figure S2: Essential gene analysis, Figure S3: Cas9-efficiency test.
