# Supplementary material for: A Genome-Wide CRISPR/Cas9 Screen Reveals the Requirement of Host Sphingomyelin Synthase 1 for Infection with Pseudorabies Virus Mutant gD–Pass
Source: Viruses. 2021 Aug 9;13(8):1574. doi: 10.3390/v13081574 (PMC8402627; doi:10.3390/v13081574)
Supplement: Supplementary file 1 [file viruses-13-01574-s001.zip › viruses-1309599-supplementary.pdf]

viruses

MDPI

A

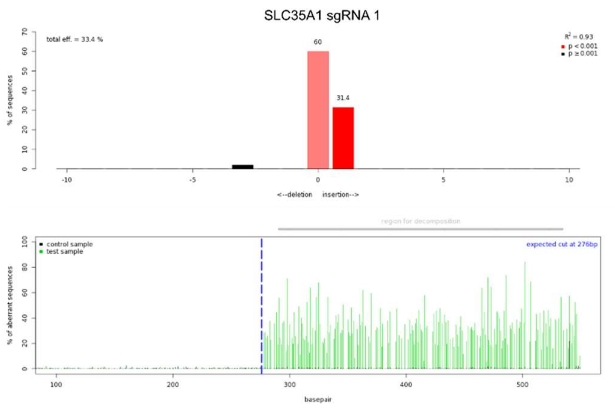

Quantification Indel Frequencies
overall efficiency = 33.4 %

	percentage	pvalue
-10	0	1
-9	0	1
-8	0	1
-7	0	1
-6	0	1
-5	0	1
-4	0	1
-3	2	0.0019
-2	0	1
-1	0	1
0	60	0
1	31.4	0
2	0	1
3	0	1
4	0	1
5	0	1
6	0	1
7	0	1
8	0	1
9	0	1
10	0	1

B

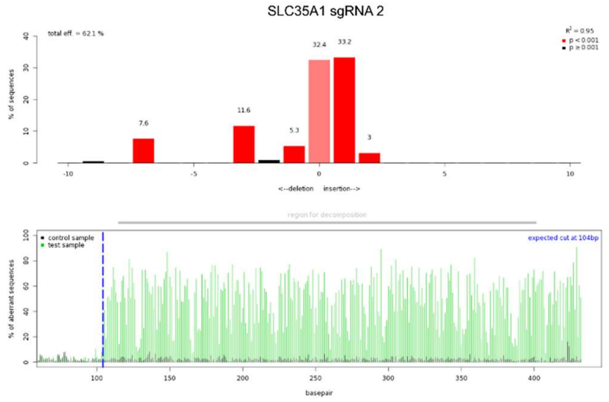

Quantification Indel Frequencies
overall efficiency = 62.1 %

	percentage	pvalue
-10	0	1
-9	0.5	0.23
-8	0	1
-7	7.6	7.9E-90
-6	0	1
-5	0	1
-4	0	1
-3	11.6	1.3E-209
-2	0.8	0.025
-1	5.3	3.5E-45
0	32.4	0
1	33.2	0
2	3	3.1E-16
3	0	1
4	0	1
5	0	1
6	0	1
7	0	1
8	0	1
9	0	1
10	0	1

C

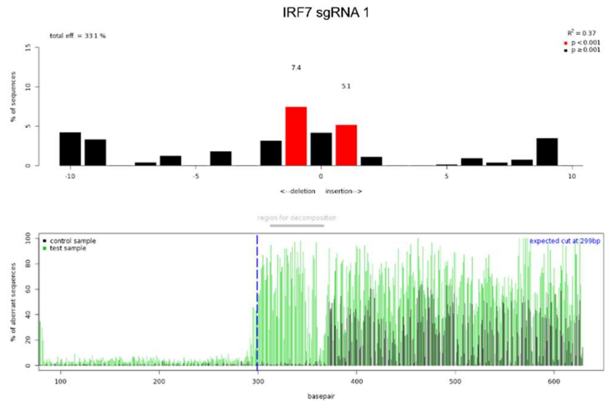

Quantification Indel Frequencies
overall efficiency = 33.1 %

	percentage	pvalue
-10	4.2	0.0049
-9	3.3	0.026
-8	0	1
-7	0.4	0.81
-6	1.2	0.4
-5	0	1
-4	1.8	0.24
-3	0	1
-2	3.1	0.029
-1	7.4	2.8E-07
0	4.1	0.0033
1	5.1	0.00028
2	1.1	0.45
3	0	1
4	0	1
5	0.1	0.95
6	0.9	0.51
7	0.4	0.79
8	0.7	0.6
9	3.5	0.0091
10	0	1

Figure S1. TIDE analysis of gene regions. In order to analyze the efficiency three sgRNAs were chosen, which target host cell factors previously described to be involved in influenza virus infection. The sialic acid transporter SLC35A1 was targeted by two sgRNAs (A and B), while in addition the transcription factor interferon regulatory factor 7 (IRF7) was targeted by one sgRNA (C). After transduction of cells with these sgRNAs, the genomic region surrounding the cutting site was amplified from both, transduced and non-transduced cells and sequences were analyzed using the web tool TIDE (http://shinyapps.datacurators.nl/tide/). Each position in the sequence trace is generally represented by a predominant nucleotide signal with minor signals from the other three

nucleotides considered as background. The percentage of these aberrant nucleotides is plotted along the sequence trace of the control (black trace) and the experimental sample (green trace). Cutting efficiencies with the generated insertion and deletion (InDel) mutations are illustrated for each sgRNA.

A

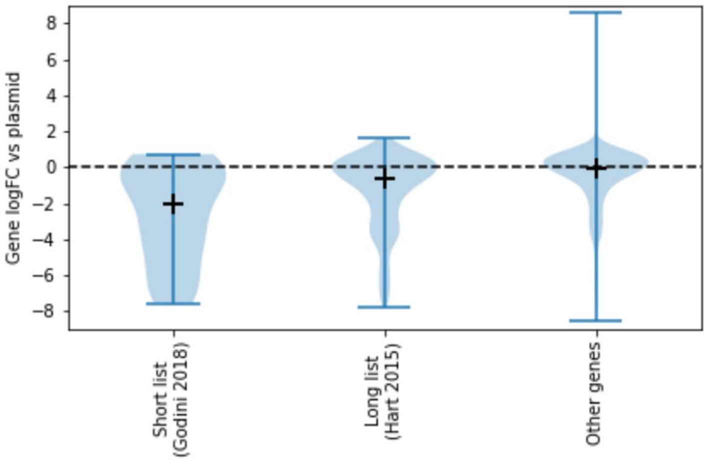

B

screening replicate	essential gene list	number of genes	permutation-based p-value	median LFC	median LFC in non-essential genes
1	short list [Godini et al., 2018]	47	<1x10^{-6}	-2.037	-0.062683
	long list [Hart et al., 2015]	1,308	<1x10^{-6}	-0.593	
2	short list [Godini et al., 2018]	47	<1x10^{-6}	-0.871	-0.016561
	long list [Hart et al., 2015]	1,308	<1x10^{-6}	-0.329	
3	short list [Godini et al., 2018]	47	0.000287	-2.8747	-1.5245
	long list [Hart et al., 2015]	1,308	<1x10^{-6}	-1.9456	

Figure S2. Essential gene analysis. To validate the library performance an essential gene analysis of the sequencing results from control cells was performed. Control cells were generated by transduction of PK15 cells with the porcine SsCRISPRko.v1 library. sgRNAs targeting genes which are essential for cell survival drop out from the integrated sgRNA pool during selection and expansion. By comparing the read counts of the control cell pool to the input plasmid, genes were ranked by fold change (FC). (A) Shows a violin plot presenting the kernel density estimate of the distribution of gene-level log FC values for essential versus non-essential genes, for one representative screen. Median log fold change is shown by the '+' symbol. The statistical details as Gene Set Enrichment Analysis (GSEA) permutation-based p-values, median log FC of essential and of non-essential genes (19,291 genes) of all three screens are shown in (B). Essential genes were considered significantly depleted in the transduced cells as shown by enrichment in higher-ranked genes, ranked in ascending order of fold change, in GSEA and median FC < 0.

.

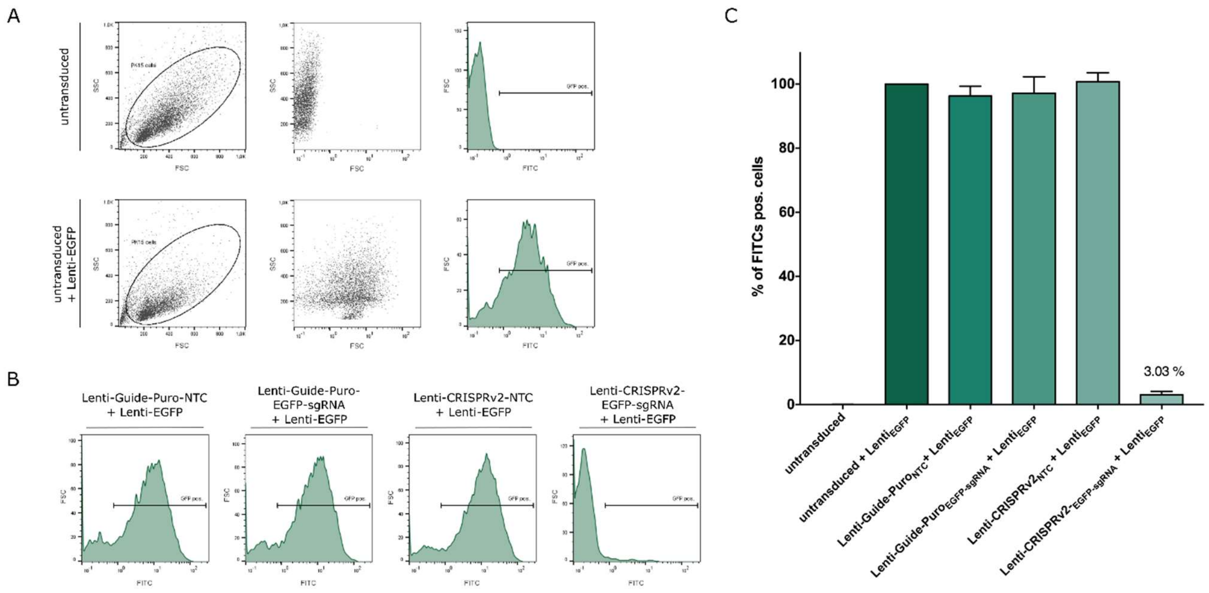

Figure S3. Cas9 efficiency test. Before starting the genome-wide screening, the suitability of the porcine kidney epithelial PK15 cell line was tested for Cas9 efficiency upon transduction with plentiCRISPRv2. For quantification, the EGFP-negative cell population was identified, gated via a corresponding non-transduced control and analyzed by flow cytometry using a MACS Quant flow cytometer. The gating strategy can be found in (A) with the examples of the untransduced or Lenti-EGFP-transduced controls. In Figure (B) the histograms show the different EGFP expression either after transduction with an empty backbone (non-targeting control, NTC) or with a backbone containing a sgRNA against EGFP. Lenti-Guide-Puro backbone, missing the genetic information for Cas9, was included as negative control. After subtraction from the total cell population the number of EGFP-positive PK15 cells was determined (C). The percentage of FITCs positive cells is depicted as mean value with corresponding standard deviations. Shown are representative dot blots/histograms from flow cytometry of five independent experiments.
